# Öffentliche Mobilität und neue Formen der Governance: das Beispiel Volksentscheid Fahrrad

**DOI:** 10.1007/978-3-658-32106-2_6

**Published:** 2020-10-24

**Authors:** Dirk von Schneidemesser

**Affiliations:** TU Berlin, Berlin, Germany; IASS Potsdam, Potsdam, Deutschland

## Abstract

Der Anstoß für Deutschlands erstes Fahrradgesetz kam aus der Zivilgesellschaft. Der Impuls entstand aus dem weiterhin ungeklärten Konflikt zwischen der Autofixierung der konservativen deutschen Verkehrspolitik und dem progressiven Wunsch nach einer nachhaltigen Mobilität, die sich an den Bedürfnissen der Bürger*innen orientieren soll und eine Umverteilung des Straßenraumes zugunsten des Radverkehrs erforderte. Dass Verkehr nachhaltiger gestaltet werden muss, war Konsens. Über das Wie gingen die Meinungen stark auseinander.

## Einleitung

Der Anstoß für Deutschlands erstes Fahrradgesetz kam aus der Zivilgesellschaft. Der Impuls entstand aus dem weiterhin ungeklärten Konflikt zwischen der Autofixierung der konservativen deutschen Verkehrspolitik und dem progressiven Wunsch nach einer nachhaltigen Mobilität, die sich an den Bedürfnissen der Bürger*innen orientieren soll und eine Umverteilung des Straßenraumes zugunsten des Radverkehrs erforderte. Dass Verkehr nachhaltiger gestaltet werden muss, war Konsens. Über das Wie gingen die Meinungen stark auseinander.

Traditionell liegt in Deutschland der Schwerpunkt des Straßenverkehrsrechts darauf, die Verhältnisse zwischen Kraftfahrzeugen untereinander und zwischen Kraftfahrzeugen und anderen Objekten zu regeln. Dem stellt Schwedes (2014) drei konträre Sichtweisen entgegen: erstens, dass es einen breiten gesellschaftlichen Konsens über die Notwendigkeit einer integrierten Verkehrspolitik gebe. Zweitens, dass der Ansatz der integrierten Mobilität keineswegs neu oder überraschend sei. Und drittens, dass dieser Ansatz – trotz Konsens und gängiger Verbreitung – kaum umgesetzt werde. Pucher und Buehler (2017) weisen darauf hin, dass der Radverkehr sowohl von der Wissenschaft als auch von Verkehrsplaner*innen jahrzehntelang vernachlässigt wurde, obwohl er eine große Rolle bei der Bewältigung der Mobilitätsherausforderungen spielen kann.

Diese Beobachtungen deuten auf eine Diskrepanz zwischen Anspruch und Wirklichkeit hin. Die daraus resultierenden Spannungen zwischen Politik und Zivilgesellschaft bildeten den Auslöser für jene zivilgesellschaftliche Initiative, die den Prozess zur Verabschiedung des Berliner Mobilitätsgesetzes – und damit auch des ersten Fahrradgesetzes – herbeiführte: Die Initiative Volksentscheid Fahrrad (VEF). Wie andernorts in Deutschland besteht auch in Berlin in weiten Teilen des politischen Spektrums Konsens darüber, dass der motorisierte Individualverkehr auf den ÖPNV und das Fahrrad verlagert werden soll (CDU Berlin 2020; FDP Berlin 2016; SPD, Die Linke, Bündnis 90/Die Grünen 2016). Trotz dieser Absicht bleibt Berlin bis heute in Bezug auf seine Verkehrsgestaltung eine autogerechte Stadt. Knapp 60 % der Verkehrsflächen in Berlin sind dem Kraftverkehr gewidmet, obwohl dieser nur etwa 25 % der Wege ausmacht – Tendenz sinkend. Dem Radverkehr hingegen werden nur 3 % der Verkehrsflächen zugestanden, obwohl er bereits für 18 % der zurückgelegten Wege aufkommt – Tendenz steigend (Gerike et al. 2020; SenUVK 2019; Agentur für clevere Städte 2014).

Aus dieser Diskrepanz zwischen Anspruch und Wirklichkeit hat sich in der Berliner Zivilgesellschaft der Wunsch nach Veränderung und einer neuen Verkehrspolitik entzündet. Berlin ist Deutschlands größte Stadt und verfügt als Stadtstaat über Kompetenzen der Landesgesetzgebung. Diese Kompetenzen sowie die in der Berliner Verfassung verankerte rechtliche Grundlage für eine Volksgesetzgebung boten die strukturellen Rahmenbedingungen für einen verkehrspolitischen Wandel mit Strahlkraft und Vorbildfunktion über Berlin hinaus.

In diesem Kapitel skizziere ich zunächst den verkehrspolitischen Kontext, bevor ich auf den konkreten Fall von Mobilitätsgovernance in Deutschland am Beispiel des VEF eingehe. In diesem Zusammenhang werden Formen der Kollaboration im Feld der Verkehrspolitik erläutert. Anschließend werden die Rollen der beteiligten Akteure im Bereich der Mobilitätssteuerung im Zusammenhang von VEF und dem Mobilitätsgesetz beleuchtet und mit Beispielen veranschaulicht. Schließlich werden Vorschläge für einen neuen Modus der Zusammenarbeit formuliert, um die gegenwärtigen kollaborativen Konstellationen mit einem zivil-orientierten kollaborativen Modus zu ergänzen.

## Der Weg zum Mobilitätsgesetz

### Übergeordnete Zusammenhänge

Die wirtschaftliche Bedeutung der Automobilindustrie führte dazu, dass das Fahrrad – trotz langer Tradition – von der deutschen Politik vernachlässigt wurde (Oosterhuis 2016), anders als in den Nachbarländern Dänemark und Niederlande. Das alltägliche Radfahren wurde aber auch nicht völlig ausgeblendet wie z. B. in Italien oder Frankreich. Dies führte zu einer Situation, in der das Fahrrad als Verkehrsmittel zwar normalisiert, aber gleichzeitig in politischer Hinsicht marginalisiert wurde. Als Anfang der 2000er-Jahre verschiedene Faktoren (politische Bedenken hinsichtlich des Klimawandels und Finanzkrisen sowie kontextbezogene Faktoren wie Bevölkerungswachstum, Urbanisierung und Digitalisierung) zusammenkamen, die die Dominanz des Automobils in der Wirtschafts- und Verkehrspolitik hätten infrage stellen können, war das Auto schon lange fest verankert (Schwedes 2017, 2011). Die korporatistischen Konstellationen, die die Governanceprozesse in Deutschland prägen (Lijphart 1999), stabilisierten die politische Relevanz des Automobils. So sind die beiden zentralen Akteursgruppen bei der bundespolitischen Verkehrsgovernance – Industrie- und Wirtschaftsverbände sowie Gewerkschaften – wirtschaftsorientierte Akteure und stark in der Automobilindustrie oder verwandten Branchen verwurzelt.

Diese wirtschaftsorientierten Strukturen verdrängten potenzielle zivile Partner*innen aus Governanceprozessen. Darüber hinaus war die Bedeutung zivilorientierter Partner durch ihre begrenzten Ressourcen weiter eingeschränkt, insbesondere im Vergleich zu Industriepartnern wie der Automobilindustrie oder den Gewerkschaften mit eigenen Finanzierungsquellen und entsprechend professioneller Organisation. Und sogar innerhalb der Zivilgesellschaft verfügten automobilindustrienahe Gruppen wie der ADAC (Allgemeiner Deutscher Automobil-Club) über weitaus mehr Ressourcen als ihr fahrradorientiertes Pendant, der ADFC (Allgemeiner Deutscher Fahrrad-Club) (Schwedes 2011). Diese Gruppen, allen voran der ADFC, waren stärker in zivilen Strukturen verankert als Ihre auto-orientierten Pendants. Sie konzentrierten sich weniger auf den Rennsport und hatten weniger Kontakte zur Fahrradindustrie als der ADAC zur Autoindustrie, sodass insgesamt deutlich weniger finanzielle Unterstützung zur Verfügung stand (Oosterhuis 2016). Das führte dazu, dass sich auto-orientierte Interessengruppen mit professionellen Strukturen und wirksamen Finanzierungsmöglichkeiten durch höhere Professionalität und Zuverlässigkeit als bessere Partner*innen positionieren konnten. Dieses Gefälle besteht noch heute.

Für den VEF und die Berliner Fahrradpolitik bedeutete dies aber auch, dass bereits etablierte Netzwerke, Strukturen und Prozesse als potenzielle Partner bzw. Ressourcen bereitstanden: Die etablierte Lobby, vertreten vor allem durch den ADFC, aber auch durch den VCD (Verkehrsclub Deutschland) und andere Organisationen.

### Der spezifische Zusammenhang: Fahrradpolitik in Berlin

In Anbetracht der oben beschriebenen Zusammenhänge überrascht es nicht, dass die Hürden für eine Umverteilung von Ressourcen im Sinne des Radverkehrs weiterhin sehr hoch sind, auch wenn die Vorteile des Radverkehrs für Alltag und Politik (als Antwort auf klima-, gesundheits-, verkehrs- sowie wirtschaftspolitische Herausforderungen) immer offensichtlicher geworden sind. Denn obgleich es jahrzehntelang Bekenntnisse eines breiten Spektrums von Politiker*innen zu einer fahrradfreundlichen Politik gab, waren die politischen Akteure nicht zur Neuordnung bestehender Prioritäten bereit. Falls die Forderung nach mehr Radverkehr in aufrichtiger Absicht erfolgte, führte der daraus resultierende Konflikt mit den Privilegien des Automobils zu Untätigkeit und setzte seine Privilegierung fort. Mit anderen Worten: Wann immer es um die konkrete Umsetzung der Förderung des Radverkehrs ging, wurde diese durch mangelnde Bereitschaft zur Umverteilung des Verkehrsraums vom motorisierten Verkehr hin zum Fahrradverkehr verhindert.

Obwohl es aufgrund der Teilung Berlins schwierig ist, belastbare Zahlen für das gesamte Stadtgebiet zwischen 1949 und 1990 zu finden, gibt es Hinweise darauf, dass der Anteil des Radverkehrs am Gesamtverkehr 1950 im Vergleich zu 2004 doppelt so hoch war (SenUVK 2019). Dieser Rückgang der Fahrradnutzung in Berlin in der zweiten Hälfte des 20. Jahrhunderts ist großenteils auf die wachsende Bedeutung des Autos zurückzuführen. Sogar innerhalb des relativ begrenzten Gebiets von West-Berlin wurden für den innerstädtischen Verkehr Autobahnen gebaut.

Die Fahrradlobby verfolgte in den 1990er und 2000er Jahren mit verschiedenen Maßnahmen die Strategie, Radfahrende deutlich sichtbar in das Blickfeld der Autofahrenden zu bringen. Dazu gehörten aufgemalte Linien als Radstreifen auf der Straße und Überzeugungsarbeit, nach der Radfahrende in der Mitte von Fahrbahnen fahren sollten. Hauptargument für diese Maßnahmen war, dass Radfahrende im direkten Blickfeld von Autofahrenden sicherer seien, weil die Autofahrenden sie dann wahrnehmen und so Kollisionen vermeiden würden. Die Fahrradlobby war mit dieser Strategie insofern erfolgreich, als dass die Zahl der gemalten Radwege (Radschutzstreifen und Radfahrstreifen) in Berlin ebenso zunahm wie die Zahl der Radfahrenden (mit Ausnahme einer Stagnationsperiode zwischen 2000 und 2005). Dennoch blieb die Bedeutung des Fahrrads als Verkehrsmittel für den Alltag aus politischer Perspektive gering und der Anteil des Radverkehrs stieg erst 2009 deutlich über 10 % (SenUVK 2019).

Trotz der steigenden Nutzung des Fahrrads und Absichtserklärungen seitens der Politik, die Fahrradinfrastruktur auszubauen (siehe z. B. die Radverkehrsstrategie für Berlin (SenStadtUm 2013)), blieben die Berliner Straßen und die Verkehrspolitik weiterhin auto-orientiert. Der Widerstand gegen Veränderungen in der Verkehrspolitik fand 2013 öffentlichkeitswirksam seinen Höhepunkt, als die damalige Berliner Regierung die Bürgerinnen und Bürger zu einem Radsicherheitsdialog einlud. Fast 30.000 Berlinerinnen und Berliner folgten dieser Einladung und identifizierten auf der dafür eingerichteten Website über 5000 Orte, an denen sie sich als Radfahrende unsicher fühlten. Dazu schrieben sie über 4000 Kommentare (SenStadtUm 2014). Viele Radfahrende hofften, dass dieser Radsicherheitsdialog eine neue Verkehrspolitik markieren und die aus ihrer Sicht lang erwartete politische und infrastrukturelle Wende zugunsten des Fahrrads eintreten würde.

Zwei Jahre später herrschte Enttäuschung: Exakt einer von mehr als 5000 im Radsicherheitsdialog identifizierten Orte war aus Sicht der Bürger*innen von der Regierung sicherer gestaltet worden (Brückner 2016). Teile der Fahrradgemeinschaft sahen deshalb die Notwendigkeit, ihre Taktik radikal zu ändern, um Veränderungen herbeizuführen. So folgte Ende 2015 eine Gruppe langjähriger und neuer Befürworter*innen des Fahrrads dem Aufruf des Aktivisten Heinrich Strößenreuther und bildeten den Volksentscheid Fahrrad (VEF), um das Ziel einer fahrradgerechten Stadt zu erreichen (Lüdemann und Strößenreuther 2018).

### Der Volksentscheid Fahrrad

**Figure 1:**
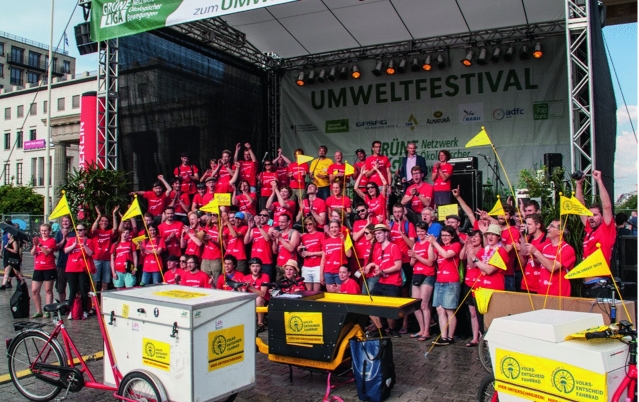

Die Anfänge des VEF waren, wie oft bei signifikanten Abweichungen vom Status-quo, von Konflikten geprägt. Diese spielten sich nicht nur zwischen VEF und politischen und administrativen Akteuren ab (Volksbegehren werden oft als direkte Herausforderung der politischen Führung wahrgenommen), sondern wurden auch innerhalb der Fahrradlobby ausgefochten. Die vom VEF gestellten und in einem Gesetzesentwurf festgehaltenen Forderungen stellten einen Paradigmenwechsel in der Verkehrspolitik dar, der sich durch drei Hauptmerkmale charakterisieren lässt: erstens, die politische Ausrichtung der Initiative; zweitens, die Neuausrichtung des Expertisebegriffs und damit auch die Funktion von Expertise und drittens, die Notwendigkeit einer neuen Beziehung zwischen der Zivilgesellschaft und den politischen und administrativen Behörden. Eine nähere Betrachtung dieser drei Merkmale erfolgt in der narrativen Beschreibung des VEF als Bewegung (Abb. 1).

#### Politische Ausrichtung: physisch getrennte Radwege und die Berliner Fahrradlobby

Zu Beginn formulierte der VEF zehn Maßnahmen, die die Stadt Berlin ergreifen sollte, um schnellstmöglich Fahrradstadt zu werden (siehe Tab. 1). Diese Maßnahmen mussten in den Entscheidungs- bzw. Zuständigkeitsbereich der Berliner Regierung fallen, realistisch und erreichbar sein. Insbesondere ein Aspekt dieser Forderungen unterschied die neue Initiative von den etablierten, reformorientierten Akteuren in der deutschen Verkehrspolitik und sogar von der Fahrradlobby: Der VEF setzte sich für eine vom Kraftverkehr physisch getrennte Fahrradinfrastruktur ein. Dieser Grundsatz schlug sich in den Zielen des VEF nieder. Das ist auch das relevanteste Merkmal, das den vom VEF eingeleiteten Paradigmenwechsel in der Verkehrspolitik verdeutlicht.

**Table 1:**
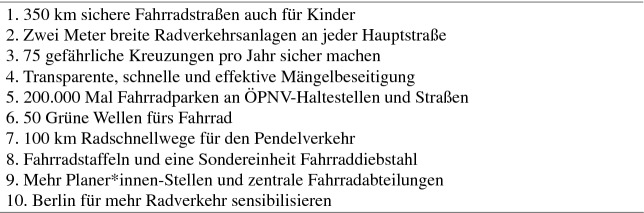

Die getrennte Infrastruktur für den Rad- und Autoverkehr wurde von Verwaltungsakteuren, Verkehrsplaner*innen und Fahrradlobbyist*innen als gefährlich abgelehnt (dies ist in gewissem Umfang auch weiterhin der Fall). Einige physisch getrennte Radwege in Deutschland leiteten Radfahrende hinter Reihen von geparkten Autos entlang. Dies bedeutete schlechte Sichtbeziehungen, die das Risiko von Unfällen aufgrund unerwarteter Begegnungen an Kreuzungen erhöhten. Die Aktivisten des VEF räumten jedoch die verbreitete Annahme aus, dass das Konzept ‚Verkehr‘ notwendigerweise den Fokus auf den Autoverkehr legen muss. Anders gesagt: Wenn Radfahrende auf einem Radweg vom Kraftfahrzeug aus nur schlecht sichtbar sind, ist nicht der Radweg das Problem, sondern es sind die parkenden Autos, die die Sicht behindern. Die in den Zielen des VEF angestrebte getrennte Infrastruktur erfordert eine massive Umverteilung des Verkehrsraums. Denn im Gegensatz zur bisher etablierten Berliner Fahrradlobby stand eine andere Personengruppe im Vordergrund der Maßnahmen: Es ging nicht in erster Linie darum, die Situation für diejenigen zu verbessern, die bereits regelmäßig Fahrrad fahren. Vielmehr sollten auch diejenigen zum regelmäßigen Fahrradfahren ermuntert werden, die dies noch nicht tun. Der VEF übertrug damit die Verantwortung für die Sicherheit von den einzelnen Radfahrenden auf die Verkehrsinfrastruktur, für welche die Politik die Verantwortung trägt. Somit wurde die Politik Hauptadressat der Forderungen des VEF.

Die Position des VEF für eine getrennte Infrastruktur wurzelte in einer damals jungen Entwicklung in Planungskreisen der USA. Dort ergaben erste Untersuchungen, dass das fehlende Sicherheitsgefühl beim Radfahren für viele ein großes Hindernis darstellt, das Fahrrad als Verkehrsmittel zu wählen (Dill und Gliebe 2008; Dill und McNiel 2012; Geller 2006). Angesichts dieser Entwicklungen war in einigen Planungskreisen außerhalb Deutschlands bereits der Ruf nach einer eigenen Infrastruktur für das Fahrrad laut geworden.

Die Debatte um getrennte Infrastrukturen war einer der ersten großen Konfliktpunkte, auf die der VEF stieß. Eine wichtige Rolle spielte dabei der Sicherheitsbegriff. Für viele etablierte Akteure im Feld der Verkehrspolitik war die objektive Sicherheit ausschlaggebend für beschlossene Maßnahmen. Für den VEF hingegen war die Erhöhung der subjektiven Sicherheit die einzige erfolgsversprechende Herangehensweise, den Radverkehrsanteil deutlich zu erhöhen. Denn so können sich auch die zahlreichen Menschen für das Rad entscheiden, die diesem Verkehrsmittel zwar aufgeschlossen gegenüberstehen, sich jedoch Sorgen um ihre Sicherheit machen (Geller 2006). Der Berliner Ortsverband des ADFC war in der Frage der getrennten Infrastruktur gespalten. Als mitgliederorientierte Organisation mit basisdemokratischer Struktur vertrat der ADFC diejenigen, die bereits Rad fuhren. Einige im ADFC argumentierten, dass Radfahrende auf der Straße oder direkt neben der Fahrbahn auf markierten Radwegen am sichersten unterwegs seien, weil sie sich so im Sichtbereich der Kraftfahrzeugfahrenden befänden. Der VEF hingegen argumentierte, dass viele Menschen es sich nicht zutrauten, mit dem Rad direkt neben dem Kraftfahrzeugverkehr zu fahren. Eine Vermittlung oder gar Lösung dieses Konflikts gestaltete sich schwierig, da die Grundannahmen sowie die daraus resultierenden Ziele dieser beiden Positionen grundverschieden waren. Die Aktivist*innen des VEF wollten möglichst viele Menschen aus dem Auto und auf das Fahrrad locken. Der ADFC wollte Sicherheit schaffen für seine Mitglieder, die das Rad bereits regelmäßig nutzten.

Damit trat ein neuer Akteur in die Öffentlichkeit, der akzeptierte und kaum hinterfragte Grundlagen der bisherigen Fahrradlobby, wie z. B. dem ADFC, ablehnte. Dennoch suchte der VEF die Unterstützung des ADFC. Denn eine Spaltung der Berliner Fahrradlobby hinsichtlich eines Fahrradgesetzes hätte dem gesetzesorientierten Ansatz geschadet. Der Konflikt zwischen beiden Standpunkten wurde auf einer Abstimmung auf der Jahrestagung des ADFC Berlin zugunsten des VEF entschieden, was jedoch den ADFC-Vorstand entzweite und zu mehreren Rücktritten führte (Hasselmann 2016). Damit war zwar die politische Ausrichtung der Berliner Fahrradlobby festgelegt, in den Ortsgruppen der deutschen Fahrradlobby jedoch sorgt diese Frage weiterhin für Konflikte.

#### Neue Expertinnen und Experten in der Verkehrspolitik

Wie im vorherigen Abschnitt erwähnt, gab es unterschiedliche Vorstellungen über den wichtigsten verkehrspolitischen Schwerpunkt. Das Feld der Verkehrspolitik selbst wird im Allgemeinen eher von technisch orientierten Experten und technokratischen Eliten dominiert (Martens 2017), dies gilt insbesondere für die deutsche Verkehrspolitik (Schwedes 2011) sowie für verwandte Bereiche wie die Klimapolitik (Hustedt 2013). Zivilgesellschaftliche Aktivist*innen, die in Deutschland Zugang zu verkehrspolitischen Gremien finden, passen sich häufig an und übernehmen diese technischen Tendenzen. Die in der deutschen Verkehrspolitik akzeptierte Expertise nimmt daher überwiegend eine vor allem technische Perspektive ein. Dies wiederum erschwert eine Berücksichtigung bürgerschaftlicher oder zivilgesellschaftlicher Expertise in politischen Prozessen.

Der VEF drängte darauf, dass andere Arten von Expertise als Grundlage für eine neue Verkehrspolitik verwendet werden sollten, die über den gewohnten Horizont hinausreichten (Schneidemesser et al. 2018). Denn es standen kaum quantitative Daten aus Deutschland zur Verfügung, mit denen sich die vom VEF geforderten Veränderungen unterstützen ließen. Diese Situation bedeutete eine Zäsur in der Herangehensweise und zeigte das Dilemma auf, eine Änderung der etablierten Verkehrspolitik in Deutschland herbeizuführen: Da die geforderten infrastrukturellen Maßnahmen in Deutschland kaum vorhanden waren, ließ sich deren Wirksamkeit kaum mit Zahlen aus Deutschland belegen.

Diese paradoxe Situation unterstreicht den Paradigmenwechsel, den der VEF einläutete. Der verfolgte Politikwechsel war radikaler als alle Forderungen, die aus dem Bereich der etablierten Verkehrspolitik selbst kommen konnten. Vor Auftreten des VEF zeigten sich häufig Probleme, wenn Spannungen nicht angegangen wurden, die durch strukturell bedingte blinde Flecken verursacht wurden. Der oben beschriebene Radsicherheitsdialog war ein Versuch der Berliner Regierung, die Rolle der Bürger*innen als Expert*innen für ihre Stadt anzuerkennen. Allerdings waren die Verkehrspolitiker*innen nicht in der Lage, den Input der bürgerschaftlichen Expert*innen in die Praxis zu integrieren. Die Informationen wurden als nicht relevant abgetan. Denn die Bürger*innen waren gebeten worden, anzugeben, wo sie sich unsicher fühlten. Die subjektiven Angaben und daraus formulierten Verbesserungsvorschläge widersprachen der von den Entscheider*innen erwarteten Objektivität. Die Erkenntnisse des Dialogs wurden planungskulturell wegmarginalisiert. Für fachfremde Vorschläge, die auf subjektiven Erfahrungen mit der Infrastruktur basierten, war kein Erfassungsprozess vorgesehen.

Für zahlreiche Aktive in der VEF-Initiative war genau diese Enttäuschung über die Unfähigkeit der Regierung, auf die von den Bürger*innen identifizierten Probleme zu reagieren, eine Motivation, den Politikwechsel mithilfe eines Volksentscheids zu erzwingen. Die Abwehrreaktionen der verkehrsplanerischen und politischen Expert*innen weisen auf die Hindernisse hin, die einer Öffnung für andere Formen der Erkenntnisgewinnung entgegenstehen. Dies galt sowohl für die politischen Akteure (Liebigt 2016) als auch für Verwaltungsakteure (Kunst 2018).

Die ehrenamtlichen Aktivist*innen vom VEF hatten 2016 einen entsprechenden Gesetzesentwurf geschrieben. Dieser wurde zunächst von den etablierten Expert*innen diskreditiert und abgelehnt. So war ein anderer Weg im politischen Prozess notwendig. Der VEF gewann die öffentliche Sympathie und Unterstützung durch direkte Appelle an die Öffentlichkeit, die für die Forderungen sensibilisiert wurde.

**Figure 2:**
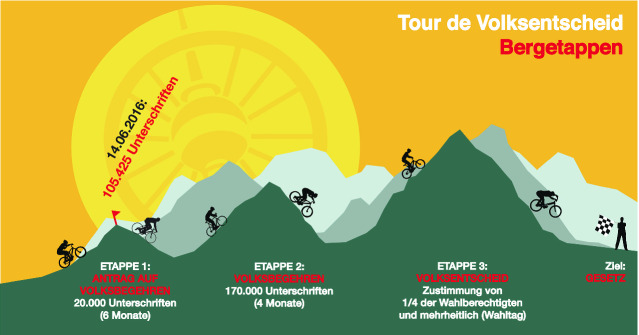

Für eine Volksabstimmung im Land Berlin musste die Initiative einen Vorschlag für eine Gesetzesänderung oder einen Gesetzesentwurf vorlegen und dann – in einem ersten Schritt – innerhalb von sechs Monaten 20.000 Unterschriften sammeln. So müsste sich das Berliner Abgeordnetenhaus mit dem Vorschlag befassen und den nächsten Schritt freigeben: Eine weitere Unterschriftensammlung, bei der dann jedoch innerhalb von vier Monaten 170.000 Unterschriften zu erzielen waren (siehe Abb. 2). Bei Erreichen dieser Vorgabe wäre das Parlament verpflichtet, den Vorschlag erneut zur Kenntnis zu nehmen und zudem eine Volksabstimmung durchzuführen (für die eine einfache Mehrheit bei einem Quorum von einem Viertel der Wahlberechtigten erforderlich ist).

Der VEF ging den ersten Schritt und sammelte innerhalb von dreieinhalb Wochen mehr als 105.000 Unterschriften – fünfmal so viele wie erforderlich und in einem Bruchteil der vorgegebenen Zeit (Lüdemann und Strößenreuther 2018; Rehmet et al. 2018). Die Unterschriften wurden im Juni 2016 dem Land Berlin übergeben. Der Vorschlag für ein Fahrradgesetz wurde von einer PR-Kampagne begleitet, die dem oben beschriebenen Konflikt zwischen Befürworter*innen und Gegner*innen der Verkehrswende Öffentlichkeit verschaffte. Damit wurden der Radverkehr und das Fahrradgesetz wichtige Themen bei der Wahl des Berliner Abgeordnetenhauses im September 2016. Der Initiative war es gelungen, die Tür für zivilgesellschaftliches Engagement in der Verkehrspolitik aufzustoßen: Wenn die politischen Akteure den ‚neuen‘ Expert*innen weiterhin die Beteiligung an verkehrspolitischen Entscheidungen versagte, würde die Politik kontinuierlich öffentlich mit deren Forderungen konfrontiert werden. Damit konnte der VEF alte Barrieren aufbrechen, durch die mobilitätspolitische Expertise von Seiten der Zivilgesellschaft bisher ignoriert worden war. Die neu gewählte Mitte-Links-Regierung verhandelte auf der Grundlage des vom VEF verfassten Gesetzesentwurfs mit der Initiative ein Mobilitätsgesetz, das schließlich im Juni 2018 mit dem Teil des Fahrradgesetzes in Kraft trat.

Vergleicht man den Gesetzesentwurf mit dem Mobilitätsgesetz, so zeigt sich, dass viele Passagen Wort für Wort aus dem Entwurf in das Gesetz übernommen wurden. In vielen weiteren Passagen wurden Inhalte direkt übertragen. In diesem Gesetz ist also buchstäblich festgeschrieben, dass sich zivilgesellschaftliches Wissen direkt in die Verkehrsplanung integrieren lässt. Die Mechanismen, die das Bürger*innenwissen als wertvolle Expertise im verkehrspolitischen Prozess zu marginalisieren suchten, ließen sich durch von der Öffentlichkeit unterstützte direktdemokratische Verfahren überwinden.

Diese direkte Gestaltung verkehrspolitischer Prozesse durch Bürger*innenwissen war beispiellos und stellte in zweierlei Hinsicht einen Paradigmenwechsel dar. Zum einen war der Inhalt eine radikale Abkehr von der in Deutschland praktizierten Verkehrspolitik. Zum anderen handelte es sich um eine neuartige Weise, wie die Politik betrieben, von Bürger*innen initiiert und schließlich in einem breit aufgestellten Prozess mit der Zivilgesellschaft verhandelt wurde.

#### Eine neue Beziehung zwischen Politik, Verwaltung und Bürger*innen

Während es bereits üblich ist, Beiträge von Interessengruppen in der Gesetzgebung zu berücksichtigen, war dieser direkte Initiierungs- und Verhandlungsprozess zwischen politischen Akteuren und der Zivilgesellschaft neu. Denn mit Verabschiedung des Gesetzes ist die zivilgesellschaftliche Beteiligung nicht vorbei: Im Gesetz ist die kontinuierliche Beteiligung der Zivilgesellschaft an der Weiterentwicklung der Verkehrspolitik sowohl auf Landes- als auch auf Bezirksebene (beispielsweise mit dem sogenannten FahrRat) festgeschrieben. Das bedeutet jedoch nicht, dass die Planungskultur damit schon erneuert ist, denn diesem partizipativen Organ sind keine verbindlichen Kompetenzen übertragen worden (Land Berlin 2018, S. 475). Es ist also derzeit noch unklar, ob der verkehrspolitische Entscheidungsprozess nachhaltig verändert wurde: Hat das Berliner Mobilitätsgesetz das Spiel tatsächlich verändert oder nur die Karten neu gemischt? 

Dessen ungeachtet haben zivilgesellschaftliche Akteure in Berlin und in ganz Deutschland das Thema Verkehrspolitik in die Öffentlichkeit getragen. Immer mehr Initiativen beanspruchen das Recht, an der Stadtentwicklungs- und Verkehrspolitik mitzuwirken (Rehmet et al. 2018). Aktuell gibt es bundesweit über dreißig Initiativen, die sich in verschiedenen Phasen des Verfahrens befinden, das der VEF in Berlin durchlaufen hat (Changing Cities 2020). Einige dieser Initiativen sind auf Landesebene angesiedelt, nämlich in Nordrhein-Westfalen und Brandenburg, die Mehrzahl engagiert sich jedoch auf kommunaler Ebene. Darüber hinaus ist Mobilität längst kein Thema mehr, das nur in Nischenmedien Beachtung findet: Verschiedene Zeitungen führen sogar Rubriken für Mobilitätsthemen jenseits des Autoverkehrs ein. Der in Berlin erscheinende Tagesspiegel zum Beispiel führt die Rubrik ‚Fahrrad und Verkehr‘ (Der Tagesspiegel 2020).

Die öffentliche Diskussion über Mobilität, frei von unverständlichem Fachjargon, bedeutet allerdings nicht automatisch, dass Mobilität wie andere gesellschaftspolitische Themen behandelt wird. Offene und transparente verkehrspolitische Prozesse lassen sich derzeit oft nur begleitet von großem öffentlichem Interesse und breiter Aufmerksamkeit aufrechterhalten. Sie sind also keineswegs garantiert. Die Nachhaltigkeit dieses Paradigmenwechsels in der Verkehrspolitik – eine neue Beziehung zwischen zivilgesellschaftlichen und politischen Akteuren – bleibt damit weiter ungewiss. Nur kontinuierliches und breites Interesse von Wissenschaft und Öffentlichkeit kann den erneuten Rückzug in technokratische Hinterzimmer verhindern. Somit ist die Zukunft zivil-orientierten Kollaborationen in der Mobilitätsgovernance noch nicht entschieden.

## Reflektionen über den Weg zum Mobilitätsgesetz

Auch wenn die neue Beziehung zwischen verkehrspolitischen Entscheidungsträgern und der Zivilgesellschaft ungewiss bleibt, so ist sie der vielversprechendste und notwendigste Aspekt des Paradigmenwechsels hin zu einer Politik der öffentlichen Mobilität. Wenn wir eine Mobilitätspraxis erzielen, die der Öffentlichkeit dienen soll, dann wird ein neuer Modus der Interaktion zwischen Verwaltung und Politik notwendig. Dieser kulturelle Wandel könnte sich als der wertvollste Beitrag des VEF erweisen (siehe den Beitrag von Hoor in diesem Band).

Zum Erreichen der Ziele des VEF war umfangreiches Wissen erforderlich. Dieses Fachwissen wurde in einem Prozess erworben, der Zehntausende von Stunden ehrenamtlichen Engagements erforderte (Volksentscheid Fahrrad 2018).

Wissen ist also nichts, was man besitzt oder nicht. Es wird in einem dynamischen Prozess immer wieder neu entwickelt. Dieses Verständnis von Wissen und Expertise ist etwas, das von den institutionellen Akteuren der Verkehrspolitik akzeptiert werden muss, um einen verkehrspolitischen und -kulturellen Wandel politischer Prozesse zu ermöglichen. Wie Arancibia (2016) argumentiert, ist die Auffassung von Wissen als persönliches Eigentum die Grundlage dafür, den Ausschluss vieler Wissensquellen (zum Beispiel von ganzen Gesellschaftsschichten) zu rechtfertigen. Denn so wird eine Trennung zwischen ‚Lai*innen‘ und ‚Expert*innen‘ ermöglicht. Demgegenüber beruht das für die Verkehrspolitik notwendige neue Verhältnis zwischen Bürger*innen, Politik und Verwaltung auf der Vorstellung, dass die Bürgerinnen und Bürger Expertise zu den Erfordernissen einer zukunftsfähigen Öffentlichen Mobilität einbringen können. In diesem Sinne bedarf es eines neuen Rollenverständnisses – insbesondere auf Seiten der Verwaltungsakteure. Dafür wurden bereits Wege aufgezeigt. Fischer (2000) hat vorgeschlagen, dass sich die Rolle der Verwaltung (zumindest teilweise) von der eines Expert*innengremiums zu einer Institution für die Vermittlung von Expertise wandeln soll.

### Eine neue Partnerschaft für die Mobilitätswende

#### Zivile Beteiligung als Ressource

Kern der neuen Beziehung zwischen Politik, Verwaltung und zivilen Akteuren ist eine Haltung, in der die Beteiligung als wertvolle Ressource wahrgenommen wird. Diese Haltung zeichnet sich durch die Idee aus, dass jedes Wissen eine von vielen verschiedenen Wissensformen ist. Die Verwaltungsakteure müssen daher die Kompetenz der Bürger*innen anerkennen, einordnen und produktiv anwenden können. Das wiederum versetzt sie in die Lage, konstruktiv mit ihnen zusammenzuarbeiten. Bei dieser Haltung geht es weniger darum, Expertise zu sammeln und zu hüten. Vielmehr erfordert es, das ständige Infragestellen des Verhältnisses zwischen den derzeitigen Kategorien ‚Lai*innen‘ und ‚Expert*innen‘ (oder in unserem Fall Bürger*innen und Verwaltung).

Der Wechsel von einem Selbstverständnis als Person, die über exklusives Fachwissen verfügt, zu einer Person, deren Aufgabe es ist, durch die Vermittlung und Anwendung von Fachwissen die Verwaltungsprozesse zu verbessern, wird nicht einfach sein. Nicht nur die gegenwärtigen Strukturen, in denen ausschließlich Verwaltungsakteure und diejenigen, die von Politik und Verwaltung beauftragt werden, als Akteure mit relevantem Fachwissen agieren, müssen sich verändern. Auch die Bürger*innen haben sich an diese Strukturen mit dem entsprechenden Expertisebegriff gewöhnt und müssen eine Veränderung erst annehmen: dass Wissen nicht mehr von einer Instanz besessen und gehütet wird, sondern dass es um das Teilen, Koordinieren und Moderieren von Wissen geht. Dies ist eine sehr einschneidende Neuausrichtung verkehrspolitischer Prozesse.

Das neue Verhältnis zwischen Bürger*innen und Verwaltung erfordert einen Wandel der Erwartungen aufseiten ersterer und grundlegend andere Strukturen, Schnittstellen und Arbeitsweisen aufseiten letzterer. Gegenwärtig ist es üblich, dass sich Verwaltungsakteure ausschließlich auf ihren Verantwortungsbereich konzentrieren und die Bearbeitung von Themen außerhalb ihrer unmittelbaren Zuständigkeit ablehnen. Dies ist selten auf böse Absicht zurückzuführen. Vielmehr beruht es auf Erfahrungen – es ist Teil des Ausdrucks einer gelernten Kultur. Für engagierte Bürger*innen kann es jedoch entmutigend sein, sich erst durch einen komplizierten Dschungel von Zuständigkeiten und Verantwortlichkeiten schlagen zu müssen. Im Bereich Mobilität als öffentliche Dienstleistung kann dies noch mühsamer sein, wenn man nicht nur den Zuständigkeitsdschungel der Verwaltung, sondern auch den der Dienstleister und Auftragnehmer durchblicken muss. Aktuell sind diese Verantwortlichkeiten teilweise sogar derart gestaltet, dass die Verantwortung nicht bei einem Akteur liegt, sondern in den Zwischenräumen versinkt (Bach und Wegrich 2016). Mit solchen Strukturen werden Bürger*innen absichtlich auf Distanz gehalten. Öffentliches Interesse an Mobilität wird hier nicht als Ressource, sondern als Bedrohung begriffen.

Darüber hinaus behindern weitere Faktoren den beschriebenen Wandel. In Berlin wurde die öffentliche Verwaltung etwa 20 Jahre lang einem Sparkurs unterworfen. Dies bedeutet, dass aufgrund fehlender Mitarbeiter in der öffentlichen Verwaltung die Kapazitäten ohnehin knapp sind. Es bedeutet ferner, dass die dort Beschäftigten tendenziell älter sind (Berliner Senat 2018). Ihre Ausbildung und prägenden Erfahrungen fielen in eine Zeit, in der es darum ging, a) die autogerechte Stadt zu bauen und b) kein Geld auszugeben. Jetzt stehen dieselben Personen vor einer Aufgabe, die erheblich von ihrer gelernten Praxis abweicht: Nämlich die Gestaltung der Stadt auf die öffentliche Mobilität auszurichten und die dafür erforderlichen Mittel auszugeben. Die Anpassung an diese Transformationsphase ist daher für viele Verwaltungsmitarbeitenden eine neue Herausforderung.

#### Ein neuer Kollaborationsmodus für Verwaltung und Zivilgesellschaft

Viele der in der öffentlichen Verwaltung beschäftigten Verkehrsingenieur*innen und -planer*innen beklagen, dass sie nicht genug Zeit für ihre Aufgaben hätten, weil sie ständig Anfragen und Beschwerden der Öffentlichkeit entgegennehmen und beantworten müssten. Gleichzeitig beklagen Politik und Verwaltungsleiter*innen, dass sie keine qualifizierten Fachkräfte fänden (Berliner Senat 2018), um die im Falle Berlins durch das Mobilitätsgesetz geforderten Planungs- und Bauaufgaben zu bewältigen. Die Schnittstelle für einen neuen Kollaborationsmodus müsste in der Verwaltung verankert sein. Sie müsste als spezielle Vermittlungsinstanz *(public interface)* verwaltungsübergreifend Input aufnehmen können. Der derzeitige Arbeitsmarkt wäre in der Lage, notwendige Kapazitäten für die Koordination von Wissen bereitzustellen, die Kommunikation zwischen ziviler Öffentlichkeit, Politik und Verwaltung zu bündeln und an die richtigen Akteure weiterzuleiten. So ließe sich in der Verwaltung Prozesswissen etablieren. Fachkräfte (bspw. Tiefbauingenieur*innen) könnten bei öffentlichkeitsorientierten Aufgaben unterstützt werden (siehe den Beitrag von Hausigke und Kruse in diesem Band).

Neue Strukturen, Haltungen und Aufgaben bedeuten neue Herausforderungen. Es darf nicht mehr sofort als Schwäche oder Wissenslücke wahrgenommen werden, wenn Verwaltungsakteure nicht sofort auf sich entwickelnde Probleme Antworten finden. Die Tatsache, dass die Regierung 2013 im Radsicherheitsdialog die Bürgerinnen und Bürger gebeten hat, die Identifizierung von Problemen und deren Lösung zu unterstützen, zeugt von dem Wunsch zumindest einiger Akteure in Politik und Verwaltung, eine neue Beziehung zu den Bürger*innen einzugehen. Die Aushandlung des Mobilitätsgesetzes mit dem VEF ist ein Beweis dafür, dass eine neue Art von Beziehung zur Zivilgesellschaft fruchtbar sein kann. Die Enttäuschung nach dem Radsicherheitsdialog und die Unzufriedenheit mit dem Tempo, mit der die Fahrradstadt realisiert wird, deuten aber auch darauf hin, dass sich diese neue Beziehung nicht reibungslos entwickelt. Der Wandel wird viel Zeit in Anspruch nehmen und möglicherweise immer wieder von Rückschlägen gebremst werden.

#### Schwierigkeiten auf dem Radweg zum neuen Kollaborationsmodus zwischen Verwaltung und Zivilgesellschaft

Aktuelle Beispiele für die Schwierigkeiten dieser Transformation sind der FahrRat und der Radverkehrsplan, die beide durch das Mobilitätsgesetz vorgesehen sind (MobG BE, §40). Das Mobilitätsgesetz sieht in §37 Absatz 7 MobG BE einen FahrRat auf Landesebene vor: ein partizipatives Gremium, in dem Akteure aus Politik, Verwaltung und Zivilgesellschaft zusammenkommen und diskutieren sollen, wie die gesetzlich vorgesehenen und weitere Veränderungen auf dem Weg zu einer fahrradfreundlichen Stadt umgesetzt werden können.

*Fallbeispiel FahrRat*

Der FahrRat ist kein neu zusammengestelltes Gremium unbekannter Akteure. Neu ist, dass er nun im Mobilitätsgesetz verankert ist, wenn auch ohne verbindliche Kompetenzen: „Er wirkt auf transparente und offene Verfahrensabläufe sowie die Einbindung aller Bevölkerungsgruppen durch geeignete Beteiligungsverfahren zu einzelnen Themen der Radverkehrspolitik hin. Der FahrRat wirkt bei der Erarbeitung und Fortschreibung des Radverkehrsplans mit. Er soll vor wesentlichen Entscheidungen und Planungen mit Auswirkungen auf die gesamtstädtische Ebene gehört werden“ ( MobG BE, §37). Theoretisch könnte dies als Grundlage dienen, den neuen Kollaborationsmodus umzusetzen. Dass dieser Anspruch trotz gesetzlich festgeschriebener Kollaboration nicht ohne Weiteres verwirklicht wird, zeigt ein Blick auf die gegenwärtige Lage.

Die Defizite der Umsetzung werden in der Praxis deutlich. Das erste Treffen des (gesetzlich verankerten) FahrRats fand im März 2020 statt – fast zwei Jahre nach Verabschiedung des Mobilitätsgesetzes. Die Verzögerung wurde der Landesregierung zugeschrieben, da sie seit der Verabschiedung des Gesetzes die Besetzung des FahrRats bestimmt. Vor der gesetzlichen Verankerung des FahrRats traf sich das Gremium dreimal pro Jahr einen ganzen Tag, aktuell wollen Verwaltungsakteure dreimal pro Jahr einen halben Tag lang zusammenkommen. Trotz der Tatsache, dass das Gesetz „transparente und offene Verfahrensabläufe“ fordert, wurde bei der ersten Sitzung vorgeschlagen, viele der Themen nicht öffentlich zu machen. Die Begründung lautete, dass andernfalls nur endgültig in der Verwaltung geprüfte und genehmigte Dinge zur Sprache kämen, keine Zwischenstände oder ungeprüfte Ideen (Quelle: persönliche Korrespondenz). Die aus der Sicht des Delegierten von Changing Cities e. V. wichtigen Themen wurden nicht auf die Tagesordnung genommen (z. B. Beschleunigung von Prozessen in der Verwaltung, Festlegung von Zuständigkeiten und Zeitvorgaben für wichtige Verwaltungsrichtlinien, Umsetzung der im Gesetz vorgesehenen Infrastruktur [mit Zeithorizonten] sowie die anstehenden großen Infrastrukturprojekte [mit Zeithorizonten]).

Der Umgang mit dem FahrRat lässt vermuten, dass die Verwaltungskultur noch sehr stark von einem statischen Wissens- bzw. Expertisebegriff geprägt ist. Der Umgang zeugt von einem Gefühl der Notwendigkeit, Strukturen aufrechtzuerhalten, in denen Expert*innen ein hohes Maß an Wissen exklusiv besitzen und somit ihre Machtposition begründen und verteidigen können. So steht weiterhin die Frage im Vordergrund, wer über welches Wissen verfügt, anstatt gemeinsam danach zu fragen, welches Wissen wie eingesetzt werden kann.

*Fallbeispiel Radverkehrsnetzplan*

Ein weiteres Beispiel ist die Entwicklung des Radverkehrsnetzes. Laut Mobilitätsgesetz hätte innerhalb eines Jahres nach dessen Verabschiedung ein Netzplan erarbeitet werden müssen. Dieser soll, zusammen mit anderen (ebenfalls noch ausstehenden) Verwaltungsrichtlinien, die Grundlage dafür bilden, wo welche Infrastruktur gebaut wird (Land Berlin 2018, §41). Über zwei Jahre nach Inkrafttreten des Gesetzes ist der Radnetzplan immer noch nicht erstellt.

Zivilgesellschaftliche Akteure wie Changing Cities e. V., ADFC Berlin, VCD Nordost oder BUND Berlin bildeten gemeinsam ein 30-köpfiges Team von Bürger*innen, das einen Radverkehrsnetzplan erarbeitete und Ende 2019 der Senatsverwaltung übergab (Changing Cities 2019b). Um der Verwaltung als Grundlage dienen zu können, bräuchte es entsprechende Überprüfungen und Anpassungen an die Arbeitsprozesse der Verwaltung. Und selbst ohne diese Überprüfungen hätte das Dokument zumindest als wertvoller Input genutzt werden können. Die Verwaltung schien jedoch nicht darauf vorbereitet zu sein, diesen Input zu akzeptieren und in ihre Prozesse zu integrieren. Stattdessen lässt die Verwaltung trotz des immensen zeitlichen Verzugs weiterhin ein Radnetz von traditionellen Expert*innen in einem Verfahren erstellen, das gewohnten Beschaffungsverfahren entspricht. (Die Errichtung von sogenannten ‚Pop-up-Radwegen‘ im Berliner Bezirk Friedrichshain-Kreuzberg als Reaktion auf die Covid-19-Pandemie zeigt jedoch, wie hilfreich der von der Zivilgesellschaft vorgeschlagene Radnetzplan für die Verwaltungsarbeit sein kann. Denn diese Radwege verlaufen auf den darin vorgeschlagenen Strecken).

Voraussetzung dafür, dass die Verwaltung von der Expertise der Zivilgesellschaft profitieren kann, wäre der oben beschriebene Kulturwandel. Die Rekonzeptualisierung von Wissen als Erwerbsprozess und nicht als Besitz würde die Integration des Wissens der Zivilgesellschaft in die Verkehrspolitik ermöglichen. Aufgrund der derzeitigen Strukturen und der verbreiteten Haltung in der Verwaltung werden Beiträge aus der Zivilgesellschaft wie das Radverkehrsnetz als ‚falsche Art‘ von Expertise abgelehnt. Dies erklärt auch die defensive und ausschließende Haltung gegenüber Gremien wie dem FahrRat. Mit einer neuen Haltung könnten diese Beiträge und Formate als Ressourcen angesehen werden. Denn wenn die Zivilgesellschaft in der Lage ist, einen Gesetzesentwurf zu schreiben, der die wichtigsten Elemente für ein fortschrittliches und zeitgemäßes Fahrrad- bzw. Mobilitätsgesetz enthält, dann ist das Wissen der Zivilgesellschaft durchaus auch geeignet, andere Aspekte des Umsetzungsprozesses der Verkehrswende zu fördern.

## Einbindung ziviler Akteure in verkehrspolitische Entscheidungs- und Verwaltungsprozesse

Das Ziel einer bürgerorientierten Öffentlichen Mobilität erfordert nicht nur eine neue Beziehung zwischen den regulär an der Mobilitätsgovernance beteiligten Akteuren, sondern auch die Einbindung neuer Akteure. Die korporatistischen Strukturen und etablierten Expert*innen haben die Integration von zivilgesellschaftlich verwurzelten Interessen in Entscheidungsprozesse der Mobilitätsgovernance blockiert. Der VEF und der Prozess zum Mobilitätsgesetz waren beispielhaft und in dem Sinn wertvoll, als dass sie zeigten, was erreicht werden kann, wenn die Zivilgesellschaft eine substanzielle Rolle im Prozess übernimmt.

Die vielen am VEF beteiligten Bürger*innen haben alle durch die Erfahrung des bürgerschaftlichen Engagements gelernt. Auch wenn für manche das Engagement nun beendet ist oder sie in anderen Bereichen aktiv werden, hat der Prozess doch eine große Zahl von Bürger*innen hervorgebracht, die wissen, wie sie sich verkehrspolitisch engagieren können. Changing Cities e. V., der Trägerverein des VEF, zählt derzeit mehr als 30 Städte mit Radentscheid-Initiativen, die bisher über 700.000 Unterschriften gesammelt haben. Das sind etwa 1,5 % der deutschen Wähler*innenschaft. Diese Menschen, ihre Motivation und ihre Erfahrungen bieten eine wertvolle Ressource für Politik und Verwaltung, die sich für die Gestaltung der Öffentlichen Mobilität nutzen ließe.

Die Rolle der Verwaltung ist hier von zentraler Bedeutung. Einerseits ist (sinnvollerweise) vorgesehen, dass die Verwaltung nicht von einem Tag auf den anderen, je nach politischer Stimmung, wichtige gesellschaftliche Handlungsrichtlinien über den Haufen wirft. Andererseits werden Veränderungen notwendig, wenn ein Konsens über längere Zeit besteht und sich daraus ein Prozess ergibt, der die Aufgaben der Verwaltung neu ausrichtet. Das ist mit dem Mobilitätsgesetz der Fall. Hier wäre eine entsprechende Neuausrichtung der Verwaltung erforderlich. Politikerinnen und Politiker, die an diesem beispiellosen Prozess, der zu einem neuen Gesetz führte, beteiligt waren, sind auf den Nachweis durch Verwaltungshandeln angewiesen, dass eine politische Neuorientierung tatsächlich Relevanz für das tägliche Mobilitätshandeln der Gesellschaft hat (Schwedes et al. 2018). Das zu versäumen, wäre ein fatales Signal an die Bürger*innen, die sich engagierten, an rechtliche Vorgaben hielten und sich demokratischer Institutionen und Verfahren bedienten.

### Beteiligung als vielversprechende Ressource in der Mobilitätswende

Die Veränderungen durch das Mobilitätsgesetz, dessen Initialzündung aus der Zivilgesellschaft kam, sind umwälzender als alle Entwicklungen, die durch die etablierten Akteure im Bereich der Verkehrspolitik in der jüngsten Vergangenheit angestoßen wurden (siehe den Beitrag von Kirchner in diesem Band). Dafür lassen sich noch weitere Beispiele finden:

- Das Bezirksamt Friedrichshain-Kreuzberg in Berlin kämpfte von 2018 bis 2019 um ein Konzept für die Bergmannstraße, eine beliebte Einkaufsstraße. Die Konzepte und Verkehrsversuche in der Bergmannstraße waren stets sehr umstritten. Als das Bezirksamt aber zufällig ausgewählte Bürger*innen einlud, sich an einem Planungsprozess für die Straße zu beteiligen, war das Ergebnis im Vergleich zu vorigen Vorschlägen von Aktivist*innen eine umfassendere Abkehr vom automobilorientierten Status-quo (Bezirksamt Friedrichshain-Kreuzberg 2019).
- In einer repräsentativen Umfrage antworteten nur 21 % der Bürger*innen, dass sich die Verkehrspolitik in Deutschland an den Bedürfnissen der Bürger*innen orientiere. 89 % antworteten, dass sich die Verkehrspolitik an den wirtschaftlichen Interessen orientiere (BMU 2019). Basierend auf den korporatistischen Kollaborationskonstellationen, die in der aktuellen Verkehrspolitik herrschen, ist dies nicht überraschend.

Diese Beispiele verdeutlichen eine Herausforderung und gleichzeitig eine Notwendigkeit. Die Politik muss zeigen, dass sie nicht nur auf dem Papier, sondern auch in der Praxis etwas verändern kann. Dazu ist sie auf eine Verwaltung angewiesen, die in der Lage ist, diesen Wandel gemeinsam mit der Zivilgesellschaft umzusetzen. Das wiederum bedeutet eine neue Planungskultur und einen neuen Kollaborationsmodus für die Mobilitätsgestaltung.

In diesem Kapitel wurde durch verschiedene Beispiele veranschaulicht, wie ein neuer Kollaborationsmodus gestaltet werden könnte und welche Voraussetzungen erfüllt sein müssten. Manche Veränderungen sind relativ unkompliziert wie die Einrichtung von Orten, an denen Zivilgesellschaft und Verwaltung zusammenkommen können. Eine weitere Voraussetzung ist Prozesskompetenz. Diese ließe sich in Form von Facilitator*innen oder Moderator*innen fördern, die sowohl die Zivilgesellschaft als auch die Verwaltung bei ihren Interaktionen begleiten. Es sind offene und verlässliche Kommunikationskanäle zwischen der Zivilgesellschaft auf der einen Seite sowie Politik und Verwaltung auf der anderen Seite nötig. Die Verwaltung ist auf eine politische Führung angewiesen, die Probleme offen anspricht und klare Führung durch den Transformationsprozess leistet. Hier wäre größere Transparenz seitens der Politik hilfreich: Welche Schritte werden im Hintergrund unternommen? Wo treten Probleme auf?

Für die Mobilitätstransformation ist eine breite Beteiligung erforderlich. Um die Probleme, die Formate wie den oben beschriebenen FahrRat plagen, zu überwinden, sind Offenheit und Transparenz hilfreich. Weder das eine noch das andere ist in der aktuellen politischen Logik selbstverständlich. Aber sie sind notwendig für einen offenen, bürgerorientierten Transformationsprozess und für die Beteiligung der Bürger*innen an Findung und Umsetzung der Lösung.

Gegenwärtig kann die Öffentlichkeit Politik und Verwaltung nur an der ‚auf der Straße‘ beobachtbaren Umsetzung der Mobilitätstransformation messen. Das führt zu konflikthaften Modi in der Kommunikation, die im folgenden Zitat zu beobachten sind: „Es geht also um die Verbesserung der Fahrradinfrastruktur auf ca. 3.100 von insgesamt 5.600 Straßenkilometern in Berlin. Um dieses Ziel zu erreichen, müssen, ausgehend vom Zeitpunkt der Verabschiedung des Gesetzes bis 2030, pro Tag 700 Meter Radverkehrsanlagen errichtet werden. Von diesen selbst gesetzten Zielen ist Berlin wortwörtlich meilenweit entfernt“ (Changing Cities 2019a). Der Fahrradteil des Mobilitätsgesetzes muss bis 2030 endgültig auf den Straßen von Berlin umgesetzt werden. So steht es im Gesetz. Und so ist durch die aktuelle Umsetzungspraxis eine Enttäuschung vorprogrammiert. Es wäre aber fatal, wenn Probleme in der Verwaltung eine ambitionierte Verkehrspolitik verhindern würden. Deshalb sollten Politiker*innen den Prozess offen gestalten und umsetzen. Sie sollte, wo möglich, Hilfe von den Bürger*innen annehmen und die Mobilitätstransformation integrativ gestalten.

#### Fazit

Der von der Zivilgesellschaft initiierte Prozess hatte durch Volksgesetzgebung ein Fahrradgesetz zum Ziel. Erreicht wurde viel mehr: ein deutlich umfassenderes Mobilitätsgesetz mit einem Fahrradteil. Der Fahrradteil ist in vielerlei Hinsicht besser als der Gesetzesentwurf, der von der Zivilgesellschaft geschrieben wurde, weil Menschen mit unterschiedlichen Hintergründen ihn im Verhandlungsprozess gemeinsam weiterentwickelten. Qualitativ profitierte das Gesetz davon, dass die Erfahrungen und das Wissen aus Verwaltung und Politik von einem zivilgesellschaftlichen Aufschlag komplementiert und bereichert wurden. Den Mehrwert, der dadurch entstanden ist, gilt es zu nutzen und umzusetzen.

Das ist der Kern der anvisierten neuen Beziehung zwischen Politik, Verwaltung und Zivilgesellschaft, die in diesem Kapitel vorgeschlagen wird. Natürlich bleibt es unbestritten, dass Politik- und Verwaltungsprozesse ihren eigenen Logiken folgen. Aber mit einem neuen kollaborativen Ansatz, der Partizipation als Ressource begreift, stünden diesen Akteuren mehr Wissen und diverse Erfahrungen zur Verfügung. Möglichweise wäre auch die Legitimation gefestigt.

Die unterschiedlichen Perspektiven, Wissensarten und Erfahrungen, die durch die hier skizzierte Haltung der Verkehrspolitik und Mobilitätsgestaltung zugutekommen würden, versprechen eine bessere Öffentliche Mobilität, die sich an den Bedürfnissen der Bürger*innen orientiert. Diese Haltung zu leben und inklusiver zu gestalten, bliebe natürlich nicht folgenlos. Es wäre einerseits mit der Entmachtung aktueller Expert*innen verbunden, andererseits würden alle beteiligten Akteure darin gestärkt, gemeinsam Verkehrspolitik zu gestalten und auf diese Weise einen Machtgewinn erfahren. Die Voraussetzung ist, dass die Verwaltung, aber auch die Zivilgesellschaft aktiv daran arbeiten, diverse Perspektiven, Wissensarten und Erfahrungen zusammenzuführen.

Um Öffentliche Mobilität zu gestalten, muss die breite Öffentlichkeit in dem Gestaltungsprozess eingebettet sein. Eine Haltung, die Partizipation als Ressource begreift, kann dazu beitragen. Das kann unterschiedliche Formen annehmen. Die Verhandlungen des Fahrradgesetzes zeigen ein Beispiel auf. Die Perspektivwerkstätten mit zufällig ausgewählten Bürger*innen für Kiezgestaltung wie im Fall der Berliner Bergmannstraße sind ein weiteres Beispiel dafür, wie Politik einen repräsentativen Teil der Gesellschaft in Planungsprozesse involvieren kann. Changing Cities e. V., der Trägerverein des VEFs, hat (wie das gesamte Feld der Verkehrspolitik) noch einen weiten Weg hinsichtlich Genderparität oder Inklusion von Migrant*innen vor sich. Es sind weiterhin viele Minderheitengruppen in verkehrspolitischen Prozessen nicht repräsentiert (siehe den Beitrag von Daubitz in diesem Band).

Die Forderungen des VEF basierten auf einer anderen Art von Wissen als der im Mobilitätsfeld etablierten, anerkannten. Zudem orientierten sie sich an anderen, noch zu identifizierenden Personen: Denjenigen, die in Zukunft Rad fahren werden. Dennoch blieben die Forderungen technisch orientiert. Physisch getrennte Fahrradinfrastruktur ist eine technische Antwort auf ein Problem, auf das auch kulturelle Antworten denkbar wären (siehe den Beitrag von Hoor in diesem Band). Freiwillige Aktivist*innen in der Zivilgesellschaft haben teilweise eigene blinde Flecken. Der VEF zum Beispiel gab keine Antwort auf Korrelationen zwischen höheren Mieten und höherer Lebensqualität durch getrennte Fahrradinfrastruktur. Hätte die Initiative eine größere Vielfalt von Personen einbezogen, wäre dies vielleicht anders gewesen. Inklusive Herangehensweisen können also für die Zivilgesellschaft Verbesserungspotenziale aufzeigen (siehe hierzu auch McCullough et al. 2019; Hoffmann 2016; Hoffmann und Lugo 2014).

Das Mobilitätsgesetz stellt einen Paradigmenwechsel in der Verkehrspolitik dar – nicht nur inhaltlich, sondern auch prozessual. Gesetzgebung ist eine extreme Maßnahme und sollte eine letzte Option sein, um einen Wandel herbeizuführen. In diesem Fall griff die Zivilgesellschaft zu der Volksgesetzgebung, weil die Appelle, die Wünsche und die Koalitionen der Willigen den Wandel, dessen Notwendigkeit politischer Konsens ist, nicht herbeigeführt haben. Die korporatistischen, kollaborativen Formate, die die deutsche Politik prägen, haben im Handlungsfeld Mobilität die Interessen der zivilen Bevölkerung zu lange beiseitegeschoben. Die Akteure im Feld der Berliner Verkehrspolitik waren nicht in der Lage, etwas mit den Ergebnissen des anberaumten Radsicherheitsdialogs im Jahr 2013 anzufangen.

Weil die Strukturen einen Wandel verhinderten, war eine Initiative wie der VEF erforderlich, um verkrustete Strukturen aufzubrechen. Das Spannungsfeld zwischen dem Konsens, dass eine neue öffentliche Mobilitätsgestaltung auf Basis einer Integrierten Verkehrspolitik nötig ist, und der Ablehnung dieser Idee durch Politik und Verwaltung, spitzte sich in einem Konflikt zu. Dieser Konflikt schuf eine Nische, die die Austragung dieser Spannungen ermöglichte. Es öffnete sich dadurch auch eine Gelegenheit für Politik und Verwaltung, gemeinsam mit der Zivilgesellschaft etwas Neues in einem neuen Modus zu realisieren.

Doch sind solche Konfrontationen – wenn auch gelegentlich notwendig – nicht immer der konstruktivste Weg. Eine neue Beziehung zwischen Zivilgesellschaft und Verwaltung könnte diese Kollaboration produktiver gestalten. Dies erfordert eine Planungskultur, die zivile Partizipation als Ressource begreift und Wissen nicht als Besitz betrachtet, sondern als Teil eines Prozesses. Eines Prozesses, der hoffentlich in eine inklusivere und nachhaltigere Öffentliche Mobilität führt.
